# Effects of targeting SLC1A5 on inhibiting gastric cancer growth and tumor development *in vitro* and *in vivo*

**DOI:** 10.18632/oncotarget.19479

**Published:** 2017-07-22

**Authors:** Jian Lu, Min Chen, Zhenhua Tao, Sumeng Gao, Yang Li, Yu Cao, Chun Lu, Xiaoping Zou

**Affiliations:** ^1^ Department of Gastroenterology, Nanjing Drum Tower Hospital Clinical College of Nanjing Medical University, Nanjing 210008, P.R. China; ^2^ Department of Gastroenterology, Nanjing Medical University Affiliated Wuxi Second Hospital, Wuxi 214002, P.R. China; ^3^ Department of Gastroenterology, The Affiliated Drum Tower Hospital of Nanjing University, Medical School, Nanjing 210008, P.R. China; ^4^ Department of Microbiology, Nanjing Medical University, Nanjing 211116, P.R. China

**Keywords:** SLC1A5, gastric cancer, cell proliferation, cell motility

## Abstract

**Aims:**

To investigate the oncogenic effects of SLC1A5 on gastric cancer development *in vitro* and *in vivo*.

**Methods:**

The expression level of SLC1A5 was detected in 70 gastric cancer paraffin-embedded tissues by immunohistochemistry and also was detected in gastric cancer cell lines by qRT-PCR and western blotting analysis. The effects of knockdown SLC1A5 were analyzed on cell proliferation, cell cycle, the ability of cell migration and invasion and growth signaling pathway *in vitro*. By using subcutaneous xenograft mouse, the importance of SLC1A5 expression was assessed for both successful engraftment and growth of gastric cancer cells *in vivo*.

**Results:**

SLC1A5 was up-regulated in gastric cancer tissues and was correlated with malignant features such as deeper local invasion, higher lymph node metastasis, advanced TNM stages and higher Ki-67 expression. Knockdown SLC1A5 in gastric cancer cells suppressed cell proliferation, caused G0/G1 arrest and inhibited cell invasion as well as migration partly by inactivated mTOR/p-70S6K1 signaling pathway *in vitro*. Furthermore, *in vivo* experiments indicated that suppression of SLC1A5 could inhibit relative volume of xenografted tumor.

**Conclusions:**

Our results suggested that SLC1A5 might be considered as a new biomarker and also as a potential therapeutic target in gastric cancer.

## INTRODUCTION

Gastric cancer (GC) is a multifactorial disease [1], which is a worldwide common cancer and ranks second in cancer-related deaths worldwide [2]. Tumorigenesis and tumor development depend on the reprogramming of tumor metabolism [3, 4] to a certain extent. Targeting reprogramming of tumor metabolism may ultimately contribute to better directing tumor therapy [5, 6, 7].

Glutamine, the most abundant amino acid in plasma [8], functions as a critical donor of nitrogen and carbon in cancer cells [9]. Given the increasing demand of glutamine in malignant tumors [10, 11, 12, 13], blocking the key components of glutamine metabolism to prevent glutamine uptake might contribute to preventing tumor cell growth.

Alanine-serine,-cysteine transporter 2 (SLC1A5; ASCT2) is the main glutamine transporter which determines the levels of intracellular glutamine [14, 15]. Meanwhile, the high levels of intracellular glutamine also are vital for maintaining activation of mTOR/p-70S6K1 signaling pathway [16, 17], a major regulator of cell proliferation [18, 19]. Targeting SLC1A5, which enables to reduce the levels of intracellular glutamine and affects the tumor microenvironment, has been considered as a promising development of SLC1A5-targeted therapy of various cancers, such as melanoma [13], on-small cell lung cancer [12, 20], prostate cancer [11], acute myeloid leukaemia [21], neuroblastoma [22], pancreatic ductal carcinoma [23], and triple-negative basal-like breast cancer [10].

In this study, we aimed to investigate the role of SLC1A5 in gastric cancer *in vitro* and *in vivo*. Our study showed that SLC1A5 was highly expressed in the specimen of gastric cancer patients and high expression of SLC1A5 in GC indicated poor clinicopathologic features. Moreover, knockdown of SLC1A5 inhibited GC cell proliferation, arrested cell cycle in G0/G1 phase and suppressed cell migration and invasion *in vitro*. Additionally, blocking SLC1A5 could restrain mTOR/p-70S6K1 signaling pathway. Loss of SLC1A5 also was efficient to suppress cell growth in subcutaneous xenograft mouse model. Our data suggested that SLC1A5 might be a potential therapeutic target in gastric cancer.

## RESULTS

### SLC1A5 is over-expressed in GC tumor specimens

**Figure 1:**
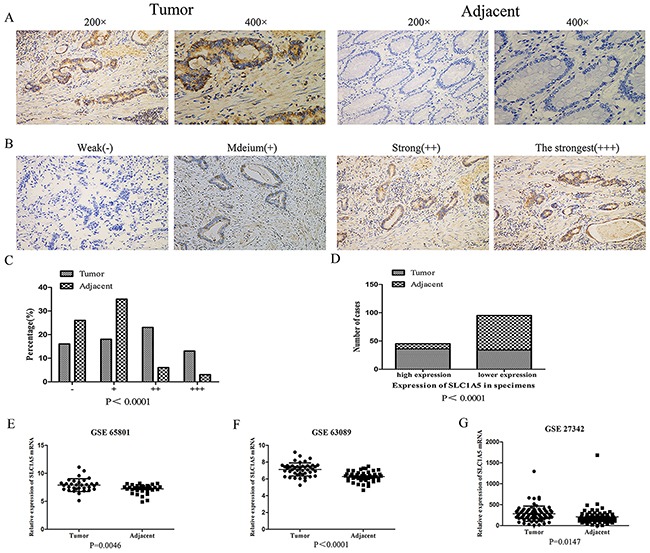
SLC1A5 was highly expressed in gastric cancers **(A)** IHC staining of human GC tissues using SLC1A5-specific antibody, as described in Methods. **(B)** Classification of samples according to the intensity of IHC staining of SLC1A5 expression. **(C-D)** The expression of SLC1A5 in tumor tissues was significantly higher than adjacent noncancerous tissues (N = 70, *p* < 0.001). **(E-G)** SLC1A5 gene expression in gastric cancer tissues (Tumor) and adjacent noncancerous tissues (Adjacent) as determined by gene expression array. Data from NCBI, GEO database GSE65801 (N=32), GSE63089 (N=45), GSE27342 (N=80).

To investigate the clinical significance of SLC1A5 in GCs, we firstly determined the expression of SLC1A5 in 70 GC patients’ samples. As shown in Figure 1A, compared to adjacent tissues, SLC1A5 highly expressed in the tumor tissues. GC samples were ranked from weak (-) to the strongest expression (+++) according to the intensity of staining (Figure 1B). The expression of SLC1A5 was higher in majority GC tissues, falling into group 3 and 4, while the expression in adjacent non-cancerous tissues was weak, falling into group 1 and 2 (Figure 1C, *P*<0.001). We further analyzed the expression levels of SLC1A5 in both GC tumor specimens and adjacent non-cancerous tissues. The expression intensity of SLC1A5 in tissues was divided into two groups: SLC1A5 lower expression group and SLC1A5 higher expression group. As observed (Figure 1D), in tumor tissues, 51.4% (36/70) of the cases showed ‘‘higher expression’’ of SLC1A5. On the contrary, in non-cancerous tissues, only 12.8% (9/70) of the cases showed ‘‘higher expression’’ of SLC1A5. The result indicated that expression of SLC1A5 in tumor tissues was significantly higher than adjacent non-cancerous tissues (*P*<0.0001). To confirm the high expression of SLC1A5 in GC, we evaluated its mRNA expression in three independent microarray data sets (GSE65801, GSE63089, GSE27342). Our analysis showed that SLC1A5 was highly expressed in GC compared with adjacent non-cancerous tissues (Figure 1E-1G).

### The expression characteristic of SLC1A5 was correlated with GC clinicopathologic features

By collecting and analyzing the data of the 70 patients, we discovered the interrelationship between the over-expression characteristic of SLC1A5 in GC and the corresponding clinicopathologic features including patients’ age, gender, tumor diameter, tumor location, histological classification, local invasion depth, lymph node metastasis, TNM stages and the Ki-67 expression. As Table 1 was shown, there was no apparent correlation between the expression of SLC1A5 and patients’ age, gender, tumor location (*P*>0.05). However, the GC tissues with higher expressed SLC1A5 had inclination towards larger tumor size (*P* < 0.05), deeper local invasion (*P* < 0.001), more lymph node metastasis (*P* < 0.05), advanced TNM stage (*P* < 0.05) and high Ki-67 expression (*P* < 0.01). Thus, these results demonstrated a significant correlation between SLC1A5 over-expression and a part of the clinicopathologic features in GC.

**Table 1:** The correlation between expression characteristic of SLC1A5 in GC specimens and GC clinicopathologic features

Clinicopathologic parameters	SLC1A5 expression		P*
	High (n = 36)	Low (n = 34)	
Age (years)			
≤60	16	13	0.5981
>60	20	21	
Gender			
Male	28	29	0.4190
Female	8	5	
Diameter (cm)			
≤5	14	22	*0.0308*
>5	22	12	
Location			
Distal third	30	25	0.3177
Middle or proximal third	6	9	
Differentiation			
Middle/well differential	6	6	0.9134
Poorly differential	30	28	
Location invasion			
T1, T2	7	20	*0.0007*
T3, T4	29	14	
Lymph node metastasis			
No	6	14	*0.0233*
Yes	30	20	
TNM stage			
I,II	7	15	*0.0263*
III,IV	29	19	
Ki-67			
Low	9	20	*0.0041*
High	27	14	

### Knock-down of SLC1A5 suppressed proliferation of GC cells and induced cell cycle arrest in GC cells

**Figure 2:**
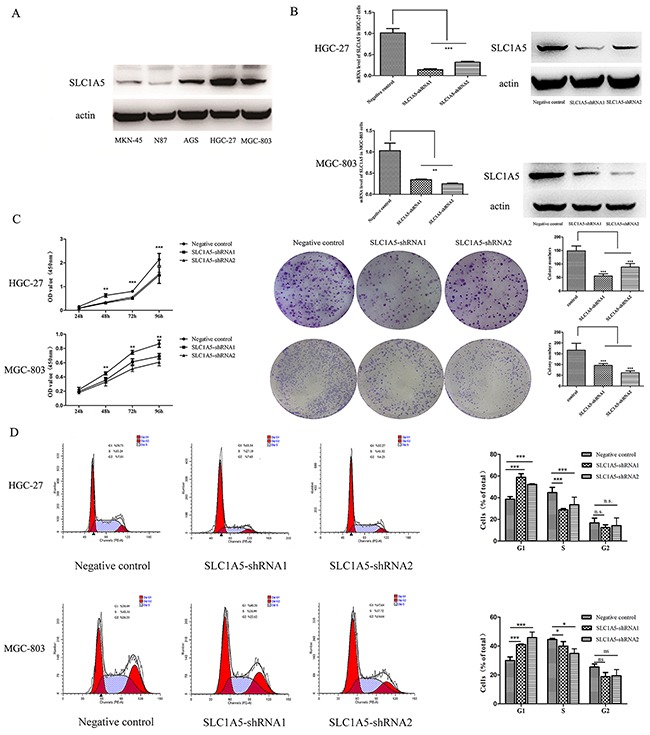
The proliferation-suppressive effect of SLC1A5 knockdown on GC cells **(A)** SLC1A5 protein expression was detected in various GC cell lines (MKN-45, N87, AGS, HGC-27 and MGC-803) by Western blotting. The protein expression of SLC1A5 was significantly higher in both HGC-27 and MGC-803cells compared with other three GC cells. **(B)** HGC-27 and MGC-803 cells were transduced with pGU6/GFP/Neo/shRNASLC1A5 vector. SLC1A5 expression significant suppressed at both mRNA and protein stage after knockdown SLC1A5(**p <0.01,***P <0.001). **(C)** CCK8 assay and clonogenic assay were used to assess the effect of SLC1A5 on cell proliferation. Data are presented as mean ± SD from three independent experiments with each running in triplicate. Unpaired student's t-test was used for the comparison between the two groups (**p < 0.01, ***p < 0.001). **(D)** Cell-cycle analysis was conducted by using Flow cytometry. Suppressed SLC1A5 significantly induced cell cycle arrest in G0/G1 phase in HGC-27 and MGC-803 cells (*p < 0.05,***p < 0.001).

We analyzed SLC1A5 protein expression in different GC cell lines (MKN-45, N87, AGS, HGC-27 and MGC-803). The results showed high SLC1A5 protein expression both in HGC-27 and MGC-803 cells (Figure 2A). By using lentiviral transduction of a SLC1A5 shRNA, we successfully performed stable transfected HGC-27 and MGC-803 cells targeted knockdown SLC1A5 (named SLC1A5-shRNA1 and SLC1A5-shRNA2) and the negative control cells. As shown in Figure 2B, the expression of SLC1A5 was significantly decreased at both mRNA and protein levels by suppressing SLC1A5. By using CCK assay and clonogenic assay, we observed a significant effect on the proliferation of HGC-27 and MGC-803 cells by knockdown SLC1A5. The results of CCK-8 assay showed a significant depression of cell growth of two GC cell lines by knock-down SLC1A5. Simultaneously, by using clonogenic assay, we found that colony formation was dramatically suppressed in SLC1A5-shRNA groups (Figure 2C). By flow cytometry analysis, we observed that knock-down SLC1A5 induced cell cycle arrest at G0/G1 phase in both HGC-27 and MGC-803 cells (Figure 2D)

### Knock-down SLC1A5 in GC cells inhibited cell migration and invasion

**Figure 3:**
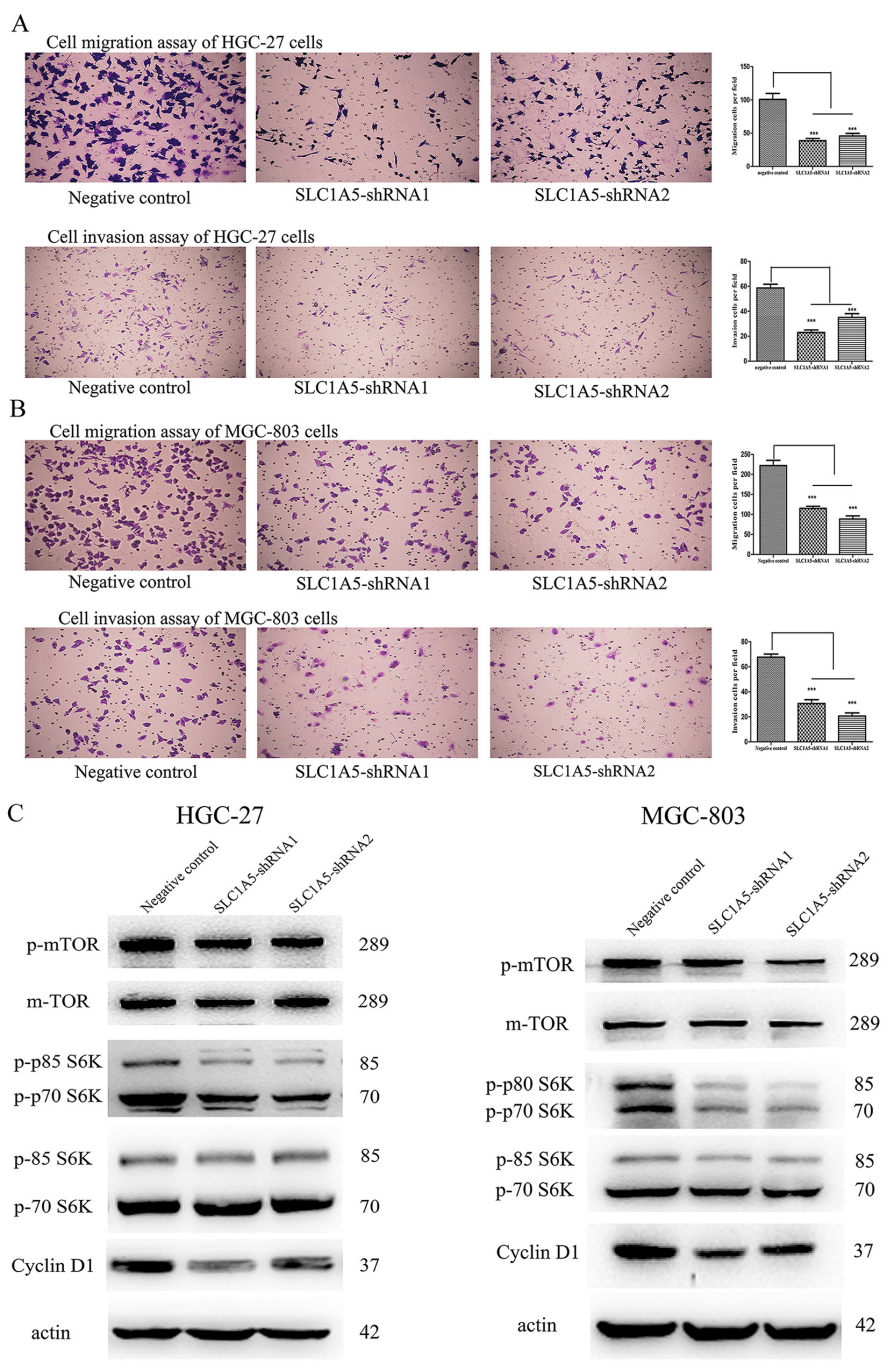
Knockdown SLC1A5 suppressed the migration and invasion of GC cells, and inhibited mTOR /p70S6K signaling pathway **(A-B)** Transwell assay was used to evaluate the efficiency of SLC1A5 on cell migration and invasion of HGC-27 and MGC-803 cells. SLC1A5 knockdown significantly decreased the numbers of invaded and migrated GC cells. The cell numbers were counted in five separate fields using light microscopy. Original magnification 400×. The data were expressed as the mean value of cells in five fields based on three independent experiments (***P<0.001). **(C)** The expression of total and phosphorylated (p-) mTOR, p70S6K and Cyclin D1 protein was detected by Western blotting after knockdown SLC1A5, with β-actin as loading control. SLC1A5 ablation down-regulated phosphorylation of mTOR and p70S6K; likewise, knockdown SLC1A5 decreased the protein expression of Cyclin D1.

We investigated the influence of SLC1A5 in the cell migration and invasion in the GC cells. As we observed in Figure 3A-3B, Knock-down of SLC1A5 could significantly inhibit the invasion and migration of HGC-27 and MGC-803 cells. These data indicated that SLC1A5 facilitated cell migration and invasion in GC cells.

### Effects of SLC1A5 on mTOR/p70S6K signaling pathway

We assessed the effects of SLC1A5 on mTOR/p70S6Ksignaling pathway. Western blot analysis indicated that the mTOR phosphorylation was decreased in HGC-27 and MGC-803 cells with SLC1A5 knockdown compared to that in negative Control. Concomitantly, the reduced phosphorylation of p70S6K was observed in the GC cells. We also found that SLC1A5 knockdown could decrease the expression of Cyclin D1 (Figure 3C). These results suggested that SLC1A5 depletion could inhibit proliferation and motility of GC cells.

### Knockdown of SLC1A5 suppressed tumor growth *in vivo*

**Figure 4:**
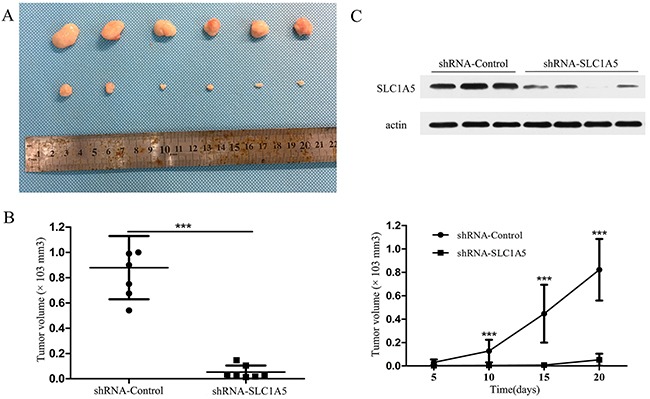
SLC1A5 knockdown suppressed tumor growth *in vivo* **(A-B)** Tumors (shRNA-Control, n=7; shRNA-SLC1A5, n=7) were harvested after 20 days, imaged and measured. Results indicated targeting SLC1A5 significantly suppressed tumor growth. **(C)** SLC1A5 was detected in HGC-27 xenograft confirmed the efficiency of knockdown of SLC1A5.

To determine whether targeting SLC1A5 repressed tumor growth *in vivo*, HGC-27 cells expressing shRNA- Control or shRNA-SLC1A5 were transduced with a lentiviral vector. HGC-27 cells expressing either shRNA-Control or shRNA-SLC1A5 were subcutaneously injected into the flank of nude mice. Knockdown SLC1A5 significantly suppressed the xenograft tumor growth when compared to the control group (Figure 4A). Results showed that shRNA-Control tumors size was significantly larger than shRNA-SLC1A5 tumors (Figure 4B, ***P<0.001). As expected, western blotting of xenograft tumors confirmed that SLC1A5 expression was significantly decreased in shRNA-SLC1A5 group compared to shRNA-Control group (Figure 4C).

## DISCUSSION

In this study, we firstly reported the oncogenic effects of SLC1A5 on gastric cancer development *in vitro* and *in vivo*. Our data showed that SLC1A5 is highly expressed in GC and is correlated with malignant features such as deeper local invasion, more lymph node metastasis, advanced TNM stages and higher Ki-67 expression. Furthermore, we demonstrated that knockdown SLC1A5 in GC cells suppressed cell growth, caused G0/G1 arrest partly by inhibiting mTOR/p-70S6K1 signaling *in vitro* and *in vivo*.

The high expression levels of SLC1A5 has been reported to be closely related to poor prognosis in many cancers [24, 25, 26, 27]. Our study also demonstrated that higher expression of SLC1A5 in GC tissues was significantly correlated with worse clinicopathologic features, such as deeper local invasion, more lymph node metastasis, advanced TNM stages and higher Ki-67 expression, which indicated poor prognosis. However, there was no significant correlation between SLC1A5 and patients’ age, gender, tumor location or the degree of differentiation. Therefore, SLC1A5 could be considered as a promising prognostic marker of GC in the future.

Considerable evidences supported that SLC1A5 was highly expressed in numbers of human malignant tumors, such as breast cancer [10], prostate cancer [11], lung cancer [12, 25], esophageal squamous cell carcinoma [24], laryngeal squamous cell carcinoma [26], tongue cancer [27] and melanoma, etc [13]. The higher levels of SLC1A5 in various types of cancer cells are considered to be essential for cell proliferation and survival via the mTOR/p-70S6K1 signalling pathway [10, 11, 12, 28]. Geldermalsen [10] reported that SLC1A5 is highly expressed in triple-negative basal-like breast cancer, and promotes cell proliferation *in vitro* and *in vivo*. Knockdown SLC1A5 inhibited cell growth, arrested cell cycle in G0/G1 stage, suppressed uptake of glutamine and then restrained mTOR/p-70S6K1 signaling pathway. This report indicated that SLC1A5 was a potential therapeutic target in breast cancers. Qian Wang [11] detected that SLC1A5 was up-regulated in prostate cancer patient samples, and promotes prostate cancer proliferation and metastasis *in vivo*. SLC1A5 suppression significantly decreased glutamine uptake, which contribute to inhibition of mTOR/p-70S6K1 signaling pathway. Thus, they suggested that targeting SLC1A5 may be a feasible therapeutic approach for prostate cancer.

Our study also showed anti-proliferation effects of down-regulation of SLC1A5 in gastric cancer cells (HGC-27 and MGC-803), and this cytostatic consequences could be explained partly by suppressing the mTOR/p-70S6K1 signaling pathway. Meanwhile, the ability of GC cells migration and invasion was significantly inhibited by down-regulating SLC1A5 *in vitro*. These data suggested that SLC1A5 could be one of optimal therapeutic targets in GC.

Our data *in vivo* further supported the results of *in vitro* experiments that SLC1A5 could be a potential targeting therapy candidate for gastric cancer. Western blotting confirmed that the expression levels of SLC1A5 protein decreased in the shRNA-SLC1A5 group compared to those in the control group. Furthermore, our results showed that suppressing SLC1A5 inhibited the relative tumor volume.

There was no doubt that there were some limitations in our experiments. The major question was that we had not confirmed whether growth inhibition of GC cells was associated with a decrease in intracellular glutamine levels by knockdown SLC1A5 *in vitro* and *in vivo*, although glutamine was an apparent major amino acid transported by SLC1A5. Moreover, we also had not detected the expression levels of mTOR/p-70S6K1 signaling pathway *in vivo*.

In conclusion, this is the first study to illuminate the role of SLC1A5 in gastric cancer *in vitro* and *in vivo*. SLC1A5 is highly expressed in gastric cancer tissues, and its high expression is correlated with poor prognosis. Our study also validated that SLC1A5 was an important glutamine transporter which promotes gastric cancer growth, and targeting SLC1A5 might have antitumor effects which partly by the inhibition of mTOR/p-70S6K1 signaling pathway. Thus, SLC1A5 may be considered as a potential biomarker and as a novel therapeutic target in gastric cancer.

## MATERIALS AND METHODS

### Patient samples

We analyzed 70 gastric cancer patients, who underwent radical gastrectomy without preoperative therapy at the affiliated Drum Tower Hospital of Nanjing University, Medical School from 2013.01 to 2013.06. The patient cohort of gastric cancer was comprised of 57 men and 13 women, with age ranging from 37 to 83 years (median, 62 years). The diameter of tumor ranged from 2cm to 8.5cm. Of the 70 gastric cancer patients, 78.6% (55/70) tumors were located in the distal third part of stomach, 21.4%(15/70) located in the middle or proximal third part of stomach. The histopathologic distribution of GC included middle/well differentiated (12/70) and poorly differentiated (58/70) GC. The depth of invasion was divided into two groups: T1/T2 (27/70,38.6%) and T3/T4(43/70,61.4%). It was showed that 28.6% (20/70) patients with no lymph node metastasis among the 70 patients, and 31.4% (22/70) were diagnosed with tumors at TNM-Stage I/II. The median value of the Ki-67 labeling index was evaluated, and the tumor cells with greater than the median value were defined as high expression. In the analyzed 70 tumor samples, high Ki-67 expression was identified in 58.6% (41/70). All gastric cancer paraffin-embedded tissues were collected to be made available for our study. Pathologic tumor-node-metastasis (TNM) stages were established using the Classification of Malignant Tumors by the International Union against Cancer (UICC) and American Joint Committee on Cancer (AJCC) system. The use of all tissue specimens for our study was approved by the Institutional Ethics Review Board of the affiliated Drum Tower Hospital of Nanjing University, Medical School.

### Cell lines and cell culture

Human GC cell lines MKN-45, N87, AGS, HGC-27 and MGC-803 were kindly provided by Department of Gastroenterology, the affiliated Drum Tower Hospital of Nanjing University, Medical School, (Nanjing, Jiangsu Province, P.R.China). These cells were cultured in RPMI-1640 (Hyclone, Logan, UT, U.S.A) medium containing 10% fetal bovine serum, (Hangzhou Sijiqing Biological Engineering Materials, Hangzhou, Zhejiang, P.R. China) in a humidified air at 37°C in 5% CO_2_. (Thermo Direct Heat CO_2_, U.S.A).

### Immunohistochemical staining and western blot analysis

Antibodies against SLC1A5 (Abcam, U.S.A) and horseradish peroxidase-conjugated secondary antibody (Abcam, U.S.A) were purchased. Immunohistochemistry analysis was carried out on tissue microarray by using antibody against SLC1A5 the manufactory instruction (1:50), and the tissues were examined respectively by two professional pathologists. The expression of SLC1A5 was considered to be positive when distinct membrane staining occurred. SLC1A5 expression was divided into four groups based on staining intensity: -, represented no staining; +, represented 1%-30% of the tumor area stained; ++, represented 30-60% stained; +++, represented more than 60% stained to a certain extent. - ~ + represented relatively lower expression and + + ~ + + + represented rather higher expression.

Cell lysates were extracted with RIPA buffer containing Protease Inhibitor Cocktail (Pierce, U.S.A), and the protein concentration was quantified using BCA Protein Assay Kit (Pierce, U.S.A). Proteins were electrophoresed and electrotransfered. The primary antibodies used were as follows: anti-SLCA5 (Abcam, USA), anti-mTOR, anti-phosphorylated mTOR, anti-p70S6K, anti-phosphorylated p70S6K (Cell Signaling Technology, Danvers, MA, U.S.A); anti-Cyclin D1(Santa Cruz Biotechnology, U.S.A).

### Stable transfection of sh-RNA suppressing SLC1A5 mRNA

pGU6/GFR/Neo vectors (synthesized by GenePharma, Shanghai, P.R.China) containing shRNA suppressing SLC1A5 mRNA and non-containing ones were used to transfect HGC-27 and MGC-803 cells. Stable transfected cells (HGC-27/SLC1A5-shRNA and MGC-803/SLC1A5-shRNA) were validated by qRT-PCR and Western blot analysis compared with the negative control cells.

### RNA isolation and qRT-PCR assay

Total RNA of HGC-27, MGC-803 cells of negative control group (empty plasmid) and SLC1A5-shRNA group cells were extracted by using TRIzol reagent (Invitrogen, U.S.A) according to the manufacture instructions. The first-strand cDNA was synthesized by using High-Capacity cDNA Reverse Transcription Kit (ABI, U.S.A). RT-primers of SLC1A5 mRNAs were designed and synthesized as follows: 5’-TGGTACGAAAATGTGGGCA-3’ (forward) and 5’-GTGCCCCAGCAGGCAGCACA-3’ (reverse) by Invitrogen Company. Real-time quantitative polymerase chain reaction (qRT-PCR) was performed according to TaqMan Gene Expression Assays protocol (ABI, U.S.A). The PCR program was set as follow: 95°C for 10 min, followed by 32 cycles of 95°C for 15 s, 60°C for 30 s, 72°C for 45 s.

### Cell proliferation and clonogenic assay

Cell viability was detected by cell counting kit-8 (CCK-8) assay (KeyGen Biotech Co. Ltd. Nanjing, Jiangsu, P.R. China). Cells transfected with the indicated shRNAs were seeded into 96-well plates at 2000 cells/well and cultured overnight at 37°C for 24, 48, 72 h. Then, 10 μl CCK-8 was added to each well and incubated for 2 h at 37°C. The absorbance was measured at 450 nm. The data were presented as mean ± SD of triplicate samples from at least three independent experiments. For the clonogenic assay, the split cells were seeded into six-well plates and cultured for 7 days. The colonies on the plates were fixed with 4% paraformaldehyde, stained with crystal violet and counted.

### Cell migration and invasion assay

Migration assay was analyzed by using the 24-Well Transwell units. Stable transfected cells (1×10^5^) suspended in 500 μl serum free medium were seeded in the upper chamber, 750 μl 15% FBS-containing medium was used as chemoattractant in the lower compartment. For the invasion assay, matrigel solution (BD Biosciences) was precoated to the upper chamber and cultured at 37°C for 3 h before cell seeding. After 24 h cultivation, Cells on the bottom surface of the membrane were fixed with 4% paraformaldehyde and stained with 0.1% crystal violet, and counted in five random fields.

### Cell cycle analysis

For cell cycle analysis, cells of negative control group (empty plasmid) and SLC1A5-shRNA group were trypsinized, then rinsed by PBS, fixed with ethanol, and handled with RNase A. The cells were analyzed by flow cytometry.

### Effects of knockdown SLC1A5 on tumor growth *in vivo*

Fourteen male immunodeficient BALB/C nude mice aged 4–6 weeks (Model Animal Research Center of Nanjing University, P.R. China) were housed in a germ-free environment, and were randomly divided into two groups (n = 7 each). Seven mice were subcutaneously injected with 3 × 10^6^ HGC-27/shRNA-Control cells resuspended in 0.1 ml of RPMI-1640 in the left ventral flanks, and other 7 mice with HGC-27/shRNA-SLC1A5 cells in the right ventral flanks. Tumor dimensions were measured once every five days with calipers. Tumor volume was estimated according to the following formula: tumor volume (mm^3^) = length (mm) ×width^2^ (mm^2^)/2. All mice were sacrificed after 20 days. Tumors were collected in lysis buffer for western blotting analysis. The *in vivo* experiments strictly obeyed the ethical principles and guidelines for scientific experiments on animals.

### Statistical analysis

All data were expressed as mean ± SD. Single factor analysis of variance test was used for comparisons among multiple groups, and *t* test was used for comparisons between two groups. *P* < 0.05 was considered statistically significant. Statistical analysis was performed using SPSS19.0 (SPSS Inc, Chicago, IL, U.S.A)
